# Internet Gaming Disorder and Nonmedical Prescription Drug Use: The Moderating Role of Student Status

**DOI:** 10.3390/ijerph23030386

**Published:** 2026-03-18

**Authors:** Steve Jacob, Kelsey A. Gately, Jonathan K. Noel, Samantha R. Rosenthal

**Affiliations:** 1 Department of Health Science, College of Health & Wellness, Johnson & Wales University, 8 Abbott Park Place, Providence, RI 02903, USAsamantha.rosenthal@jwu.edu (S.R.R.); 2 Center for Student Research & Interdisciplinary Collaboration, Johnson & Wales University, Providence, RI 02903, USA; 3 Department of Epidemiology, Brown University School of Public Health, Providence, RI 02903, USA

**Keywords:** gaming addiction, video games, prescription drug misuse, substance use, college student

## Abstract

**Highlights:**

**Public health relevance—How does this work relate to a public health issue?**

- Gaming addiction symptoms share numerous risk factors with nonmedical prescription drug use (NMPDU), and this association may be influenced by student status.
- The study emphasizes the role of educational environments in influencing behavioral and substance-related risks.

**Public health significance—Why is this work of significance to public health?**

- Higher Gaming Addiction Scale (GAS) scores may exacerbate susceptibility to other addictive behaviors, including NMPDU, and this effect may be intensified among students.
- Suggests that increases in gaming addiction symptoms may lead to meaningful increases in substance misuse risk.

**Public health implications—What are the key implications or messages for practitioners, policy makers and/or researchers in public health?**

- Clinicians should routinely evaluate gaming addiction symptoms, and early identification may reduce subsequent nonmedical prescription drug use.
- Interventions on campuses that address stress management and healthy coping methods should be considered.

**Abstract:**

Internet gaming disorder (IGD) and nonmedical prescription drug use (NMPDU) are prevalent, co-occurring concerns among young adults. Although prior research links problematic gaming and substance misuse, few studies have examined this relationship in non-college populations or whether student status modifies this association. This study examined the relationship between Gaming Addiction Scale (GAS) score and NMPDU among 1022 Rhode Island young adults aged 18 to 25. In the total sample, 44.6% identified as cisgender heterosexual female, 42.4% as sexual or gender minority (SGM), and 13.0% as cisgender heterosexual male. Multivariable logistic regression estimated the adjusted association between GAS scores and NMPDU, and an interaction term between GAS and student status was tested. Overall, 12.1% reported lifetime NMPDU. Higher GAS scores were associated with increased odds of NMPDU (adjusted odds ratio [AOR] = 1.05; 95% confidence interval [CI]: 1.01–1.09). Student status alone was not significantly associated with NMPDU; however, a significant interaction was observed between GAS and student status (AOR = 1.09, 95% CI: 1.01–1.18, *p* = 0.031). Higher GAS scores were positively associated with NMPDU, with student status strengthening this association. Findings support screening for problematic gaming, particularly among students, and integrated prevention strategies addressing both behavioral and substance-related risks.

## 1. Introduction

Internet gaming disorder (IGD), also known as video game addiction, is a significant issue in the United States (US) and reportedly affects nearly one in four young adults [1]. Currently, over three-quarters of American households own a video game console, and gaming is considered the fastest-growing entertainment industry in the world [2,3]. This popularity is driven by recent advancements in accessibility, particularly through mobile gaming and smartphone adoption, allowing for gaming experiences not limited to specific times or places [4]. Likewise, the emergence of social media games and online multiplayer games emphasize gaming as a low-barrier social activity, enabling community formation [5]. In addition, gaming serves as a form of alternative socialization that has been legitimized by the COVID-19 pandemic [6]. Video games are defined as any technology-based game requiring the manipulation of images on a visual display, including online, mobile, and casino-style games [7]. Individuals who regularly engage in video game play are commonly referred to as gamers [7]. Between 2014 and 2020, the number of active video game users globally rose from 1.8 billion to 2.7 billion, and in 2024, the video game industry is expected to produce $55.87 billion in North American revenue [8,9,10]. While surveillance studies do not routinely measure video game use and addiction, prevalence estimates are reported by large national studies. For example, IGD was reported in 24.3% of US young adults aged 18 to 25 years old [1] and 10.9% of Turkish students aged 15 to 30 years old [11]. However, prevalence was as high as 62.1% among Saudi Arabian adolescents aged 12 to 16 years old [12].

Diagnostic frameworks differ in their classification of IGD. For example, the fifth revision of the Diagnostic and Statistical Manual of Mental Disorders (DSM-5) includes IGD as a condition for further study, with the intent of establishing it as a mental disorder in subsequent DSM revisions upon further research [13]. In comparison, Gaming Disorder (GD) is formally recognized as a diagnosable condition in the 11th Revision of the International Classification of Diseases, 11th Revision (ICD-11), including both online and offline gaming [14]. Moreover, the classification of problematic gaming as a behavioral addiction is disputed, with some scholars noting the lack of symptomatology consensus, while others emphasize that the public health need justifies its inclusion [15,16].

Initial research suggests a consistent relationship between problematic gaming and substance use. In a nationally representative study of 11–19-year-olds in the Czech Republic, 20.5% of disordered gamers reported lifetime pharmaceutical misuse compared to 13.5% of non-disordered gamers [17]. Moreover, in a separate study of Czech and Slovak online gamers, 2.8% used psychoactive medications, and use was associated with an additional 6.2 to 9.8 h of gaming per week [18]. High-risk gamers also note significantly higher lifetime prevalences of polysubstance use and greater daily consumption of alcohol and tobacco compared to low-risk gamers and non-risk gamers [19]. Furthermore, in a cross-sectional study of young adults in the United States aged 18–25 years old, individuals with IGD had 1.26 times higher odds of reporting problematic alcohol use compared to those without IGD [1]. Indeed, IGD may involve similar underlying neurocognitive vulnerabilities to substance disorders, as studies suggest individuals with IGD and alcohol use disorder (AUD) share identical impairments in high motor impulsivity and poor response inhibition [20].

Risk factors for problematic gaming and NMPDU are similar. Gender differences in gaming behavior and IGD prevalence are consistently reported, as problematic gaming is typically higher in individuals identifying as males, who make up 53% of US video gamers [21]. Further, a meta-analysis of 53 studies suggested that males were 2.5 times more likely to meet the criteria for IGD compared to females [22]. Males are also associated with a higher likelihood of NMPDU [23]. Studies suggest that young adults are more likely to experience problematic gaming compared to all other older adults [24]. College students are also at a higher risk of NMPDU, as approximately 4.8% of students aged 18 to 22 years old have misused prescription stimulants in the past year compared to 3.1% of individuals with only a high school degree [25]. Among students who were prescribed stimulants, 28.4% reported taking higher than prescribed dosages [26]. Similarly, a notable percentage of college students reported easy access to prescription drugs, as 30% obtained substances from a friend [27,28].

Enrollment in college or university may moderate the association between problematic gaming and NMPDU. Individuals of student status are likely to experience greater environmental pressures, which may lead to addictive behavior in young adults [29,30]. For instance, students who report low academic performance have 2.53 times the odds of developing IGD [11], and 49.2% of undergraduates (mean age = 19.5 years) cited intentions to misuse prescription drugs, with spikes in stress significantly predicting actual misuse [31]. Furthermore, among students who engaged in nonmedical use of prescription stimulants during the past six months, 64.9% endorsed the belief that these drugs confer an academic benefit, compared with only 24.1% of non-users [29]. In addition, while college students primarily take nonmedically prescribed drugs to improve concentration [32], this behavior is tied to longer gaming durations [33]. Extensive gaming is also linked to lower social engagement and diminished academic achievement, exacerbating the risk of IGD and NMPDU [34,35,36].

Despite prior studies investigating NMPDU and IGD, no study to our knowledge has analyzed this association among non-college-specific young adult populations, nor have they examined the role of student status in the relationship between Gaming Addiction Scale (GAS) scores and NMPDU. Therefore, the primary objective of this study was to examine the association between gaming addiction symptom severity and lifetime NMPDU among young adults in Rhode Island. The secondary objective was to assess whether student status moderates this association. We hypothesized that higher GAS scores would be associated with increased odds of lifetime NMPDU and that this association would be stronger among students compared to non-students.

## 2. Methods

This study is a secondary analysis of the 2022 Rhode Island Young Adult Survey (RIYAS), which was a cross-sectional surveillance system that aimed to assess mental health status and associated behaviors in Rhode Island’s young adults.

### 2.1. Sample

The RIYAS was a self-report, de-identified, cross-sectional web-based survey implemented by the Rhode Island Department of Behavioral Healthcare, Developmental Disabilities and Hospitals (BHDDH). The 2022 RIYAS was administered between May and August 2022. Inclusion criteria were: (1) age 18–25 years, (2) residence in Rhode Island for at least part of the year, and (3) ability to complete a web-based English-language survey. The RIYAS utilized a non-probability convenience sampling strategy, recruiting participants through targeted social media advertisements and institutional outreach. Participants completed the anonymous web-based survey via a secure online platform. Upon completion, participants received a $10 electronic gift card delivered through a separate system not linked to survey responses. As this study represents a secondary analysis of an existing surveillance dataset, no a priori power analysis was conducted. All complete surveys meeting inclusion criteria (N = 1022) were included in analyses. All survey submissions had complete responses on primary study variables and were included. Participants provided electronic informed consent at the time of survey completion. The RIYAS was conducted under its own ethical approval. The present study represents a secondary analysis of a previously collected, de-identified dataset provided by BHDDH. The secondary analysis protocol was approved by the Johnson & Wales University Institutional Review Board (IRB) (Protocol RPA#220907; approval date: 11 October 2022), and all analyses were initiated only after IRB approval was obtained.

### 2.2. Measures

Prescription drug misuse is broadly defined as the use of medications at frequencies or dosages greater than prescribed or for a purpose inconsistent with that of their initial medical indication [37]. NMPDU refers to use of medications without a valid prescription or use in a manner contrary to the prescriber’s instructions. As the RIYAS survey item evaluated prescription drugs not prescribed to the participant, NMPDU is used in the present study [38].

Lifetime NMPDU, the dependent variable, was assessed through the question “Have you ever used prescription drugs not prescribed to you?” This variable was dichotomized and response options of “yes, in the past month” and “yes, more than a month ago” were both considered affirmative responses for use. This item refers to prescription medications and does not include over-the-counter drugs. The NMPDU item was designed to align with established public health surveillance definitions of nonmedical use/misuse of prescription drugs (i.e., use of prescription medication not prescribed to the respondent and/or taken for the experience or feeling it caused), consistent with the National Survey of Drug Use and Health (NSDUH) and Monitoring the Future (MTF) measures [39,40]. Similar self-report items have been widely used in epidemiologic studies of nonmedical prescription drug use among adolescents and young adults [41]. Gaming addiction symptom severity, the independent variable of interest, was assessed using the 7-item Gaming Addiction Scale [42]. The GAS assesses gaming addiction using 7 items adapted from the DSM-IV criteria for pathological gambling (i.e., salience, tolerance, mood modification, withdrawal, relapse, conflict, problems) [42]. Each of the responses were preceded by the statement “How often during the last six months …” and was recorded on a five-point Likert scale varying from “never” (coded as one) to “very often” (coded as five). Example items include “Did you think about playing a game all day long?” and “Did you spend increasing amounts of time on games?” Item responses were summed to create a total GAS score (range: 7–35), with higher scores indicating greater symptom severity. Internal consistency in this sample was high (Cronbach’s α = 0.86). Higher GAS scores indicate greater gaming addiction symptom severity, and the reliability and validity of the GAS has been previously established, with prior validation studies reporting Cronbach’s α coefficients ranging from 0.81 to 0.95 [42,43]. Student status, the moderator under study, was assessed to categorize participants as either student or not a student.

Measured potential confounders were included based on prior literature demonstrating disparities in both IGD and NMPDU use by alcohol use, cannabis use, depressive symptoms, and anxious symptoms [44,45,46].

The 10-item Alcohol Use Disorders Identification Test (AUDIT) was used to assess alcohol use severity [47]. The AUDIT is a valid and reliable assessment that includes items that assess alcohol consumption, drinking behavior, and alcohol related problems [48]. Responses are rated on 5-point Likert scales, and response frequencies vary across items. Example response options range from “never” to “daily or almost daily.” Response options for the final two items include “never;” “yes, but not in the past year;” or “yes, during the past year.” Total scores range from 0 to 40, and higher scores indicate greater alcohol use severity. In this sample, the internal consistency was good (Cronbach’s α = 0.82). Cannabis use severity was assessed via the Cannabis Use Disorders Identification Test—Revised (CUDIT-R) [49]. The CUDIT-R is a valid and reliable eight-item assessment that identifies problematic or harmful cannabis use [50]. Frequency responses vary on a five-point Likert scale. Example responses range from “never” to “daily” or “almost daily.” Response options for the final item include “never,” “yes, but not in the past 6 months,” or “yes, during the past 6 months.” Total scores range from 0 to 32, and higher scores suggest more severe cannabis use severity. Reliability in this sample was good (Cronbach’s α = 0.82). Depressive symptoms were assessed using the 10-item Center of Epidemiologic Studies Depression Scale (CES-D10) [51]. This valid and reliable scale includes 10 items that measure the frequency of past week symptoms related to depression development [52]. All responses are collected on a four-point Likert scale with response options ranging from “rarely” or “none of the time” to “most of the time.” Total scores can range from 0 to 30, and higher scores indicate greater depressive symptoms. Internal consistency in this sample was high (Cronbach’s α = 0.86). The Generalized Anxiety Disorder 7-item scale (GAD-7) was used to assess anxious symptoms [53]. A four-point Likert scale is used to collect responses, and response options range from “not at all” to “nearly every day.” Total scores range from 0 to 21 and higher scores indicate greater symptom severity. The GAD-7 is a valid and reliable questionnaire, and internal consistency in this sample was excellent (Cronbach’s α = 0.93) [54].

Numerous sociodemographic variables (i.e., age, sex, gender, and sexuality, race/ethnicity, and social status) were included a priori as standard sociodemographic covariates in behavioral health epidemiology [55]. Sex assigned at birth (male, female, intersex), gender (woman, man, non-binary, two-spirit, different identity not listed), and sexuality (heterosexual/straight, homosexual/lesbian or gay, bisexual, don’t know, different identity not listed) were collapsed as a single variable that categorized participants as “cisgender heterosexual male,” “cisgender heterosexual female,” and any “sexual or gender minority (SGM).” SGM included anyone with sex that was intersex, anyone whose gender did not match their sex, or anyone who reported their sexuality as something other than heterosexual/straight. Race/ethnicity was collapsed as a single variable that included “White non-Hispanic,” “Black non-Hispanic,” “Asian non-Hispanic,” “Hispanic,” and “Other/Multiracial non-Hispanic” (including Native American/Alaskan Native, Hawaiian and other Pacific Islander). Social status was measured using the MacArthur Scale of Subjective Social Status, which asks participants to rank themselves compared to others in the community on a scale from worst off (coded as 1) to best off (coded as 10) [56]. The scale has demonstrated construct validity through associations with objective socioeconomic indicators (e.g., income, education) and has shown predictive validity for physical and mental health outcomes across diverse populations [57,58]. Prior research also supports its test–retest reliability and sensitivity to psychosocial gradients in health [59].

### 2.3. Statistical Analysis

Descriptive statistics were calculated for all study variables. Normality of continuous variables was assessed through visual inspection of histograms and Q–Q plots. Age, social status, AUDIT scores, CUDIT-R scores, depressive symptoms, and anxious symptoms were considered normally distributed continuous variables after assessing their distributions, although GAS was not. All others were categorical variables. The analysis then proceeded in three steps. First, bivariable statistics were computed. For bivariable tests, two-sample *t*-tests were used for normally distributed continuous variables, chi-square tests for categorical variables, Wilcoxon rank-sum test for GAS due to its non-normality, and Fisher’s exact test was used for categorical variables when a single cell had fewer than 5 observations. Simple logistic regression was also used to calculate crude odds ratios (OR) for NMPDU for all study variables. Second, multivariable logistic regression was used to estimate the adjusted odds of NMPDU with each additional unit in GAS score while controlling for age, sex, gender, and sexuality, race/ethnicity, social status, AUDIT score, CUDIT-R score, depressive symptoms, anxious symptoms, and student status. Logistic regression was selected because the outcome variable (lifetime NMPDU) was dichotomous. This approach allows estimation of odds ratios while adjusting for multiple covariates and is standard in epidemiologic studies of binary behavioral health outcomes. The reference groups in the model were cisgender heterosexual male, and White non-Hispanic. Third, an interaction term between GAS score and student status was also added to the model and tested for significance. Predicted probability plots were generated to further probe the interaction. Interaction effects were interpreted by examining both the multiplicative term and predicted probabilities across GAS levels by student status. All statistical tests were assessed at 95% confidence intervals (CI) and *p*-values ≤ 0.05 were used to determine statistical significance. All statistical analyses were conducted in Stata, version 15 [60].

## 3. Results

Among Rhode Island young adults (N = 1022), 12.1% (n = 124) reported lifetime NMPDU. The mean age was 21.3 years (SE = 0.07); 61.0% identified as White non-Hispanic, 44.6% as heterosexual cisgender female, and 42.4% as SGM. Students comprised 70.4% of the sample. The GAS was positively skewed (median = 7, interquartile range [IQR] = 4). Descriptive characteristics are shown in Table 1.

Median GAS scores differed significantly by NMPDU (*z* = −2.36, *p* = 0.018). Participants reporting NMPDU had higher mean AUDIT (*t*(1022) = −5.64, *p* < 0.001), CUDIT-R (*t*(1022) = −5.65, *p* < 0.001), depressive symptoms (*t*(1022) = −4.87, *p* < 0.001), and anxious symptoms (*t*(1022) = −4.38, *p* < 0.001) scores. NMPDU did not differ significantly by sex, gender, and sexuality (χ^2^(2) = 4.25, *p* = 0.119), race/ethnicity (*p* = 0.302), social status (*t*(1022) = −0.34, *p* = 0.731), nor student status (χ^2^(1) = 3.75, *p* = 0.053) (Table 1).

In crude models, each one-unit increase in GAS was associated with 6% higher odds of lifetime NMPDU (OR = 1.06, 95% CI: 1.03–1.10, *p* < 0.001). The direction and magnitude of this relationship was maintained in the adjusted analysis (adjusted odds ratio [AOR] = 1.05, 95% CI: 1.01–1.09, *p* = 0.017). This indicates that for each one-point increase in GAS score, the odds of lifetime NMPDU increased by 5%, holding all covariates constant. Given the GAS range of 7–35, a 5-point increase in GAS corresponds to approximately a 28% increase in the odds of NMPDU, suggesting that differences in symptom severity may translate into meaningful increases in substance misuse risk. The multivariable logistic regression model was statistically significant, χ^2^(14) = 66.78, *p* < 0.001, indicating that the predictors reliably distinguished between participants with and without lifetime NMPDU. In the fully adjusted model without interaction, student status was not independently associated with NMPDU (AOR) = 0.80, 95% CI: 0.51–1.26, *p* = 0.337). Hispanic ethnicity (vs. White non-Hispanic), higher AUDIT scores, and higher CUDIT-R scores were associated with increased odds of NMPDU (Table 2). Other covariates were not statistically significant.

**Figure 1:**
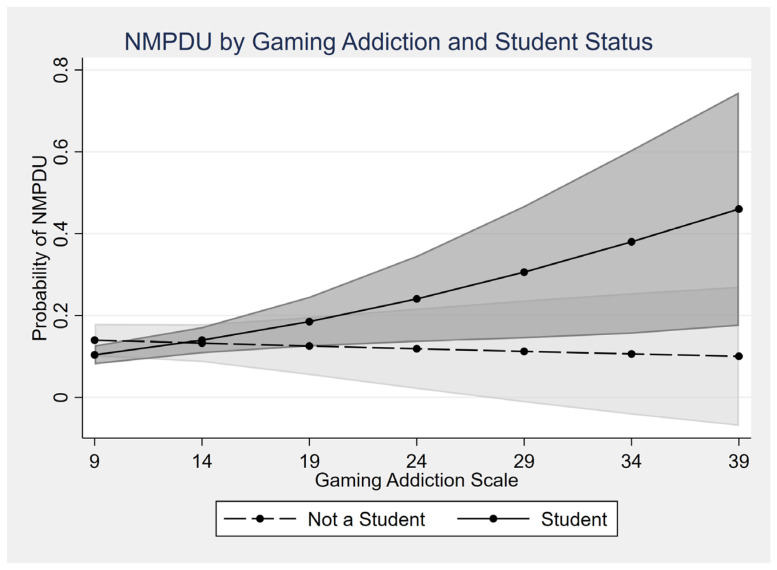
Predicted Probability of Nonmedical Prescription Drug Use (NMPDU) by Gaming Addiction Scale (GAS) and Student Status. Shaded areas represent 95% confidence intervals for each group.

The multivariable logistic regression model with interaction term was statistically significant, χ^2^(15) = 71.75, *p* < 0.001, Nagelkerke R^2^ = 0.12. When including the interaction between GAS score and student status, the interaction term was statistically significant (AOR = 1.09, CI: 1.01–1.18, *p* = 0.031) (Figure 1). This indicates that the association between GAS score and NMPDU was 9% stronger per unit increase in GAS among students compared to non-students. In other words, the slope of the relationship between gaming severity and NMPDU is steeper in students. For example, at higher GAS levels (e.g., ≥20), predicted probabilities of NMPDU diverge substantially between students and non-students (Figure 1), highlighting the amplified risk among students with elevated gaming symptoms.

## 4. Discussion

This study aimed to explore and highlight the association between GAS score and NMPDU among young adults in Rhode Island, as well as examine the role of student status in the relationship between gaming addiction symptom severity and NMPDU. The results suggest that NMPDU is common among Rhode Island’s young adults, and an association between gaming addiction symptom severity and NMPDU was found. Moreover, the interaction suggests that incremental increases in gaming addiction symptom severity confer disproportionately greater substance misuse risk among students relative to non-students, indicating that academic environments may intensify vulnerability. Although the per-unit increase in odds may appear modest (5%), cumulative increases across the GAS scale represent substantial risk differentials, particularly among students.

The association of higher GAS and concurrent use of substances is consistent with previous literature and can be understood through several mechanisms [61]. First, these behaviors may share psychological risk factors, and excessive video game use and NMPDU are similarly influenced by poor emotional regulation [45,62]. The immersive nature of video games may promote an engaging environment that reduces awareness of the risks associated with reckless behaviors such as NMPDU, especially among individuals with higher GAS [63]. Second, while coping mechanisms were not measured in our study, prior literature suggests that individuals with behavioral addictions may exhibit impaired probability judgments and that use of maladaptive coping strategies is common among populations with high gaming addiction scores [64,65]. Given that gamers with higher GAS scores may experience difficulty managing coexisting depression or anxiety, these individuals may be drawn to other addictive behaviors, such as nonmedical use of prescription drugs, which offer a similar stimulating outlet for temporary relief [66]. As studies show the majority of individuals reporting misuse obtain prescription medications from peers, this may lower barriers to misuse for individuals already struggling with impulse control associated with high GAS scores [67]. Therefore, it is possible that when coupled with their decreased risk awareness, gamers with higher GAS are uniquely vulnerable and more likely to engage in NMPDU as a maladaptive coping strategy for pre-existing mental conditions [68,69].

Our study findings also underscore the moderating role of student status in the relationship between GAS score and NMPDU. In students, video game use is often characterized as a means to alleviate academic stress [70], while prescription stimulant misuse is strongly warranted by the desire to improve academic performance [71,72]. These dynamics may be reinforced in gaming subcultures that normalize substance use as a pragmatic strategy for sustained endurance, which may extend to academic contexts [18]. As illicit prescriptions are readily attainable among university campuses, students with higher GAS scores may attempt to supplement lower grades through stimulant misuse [73]. This effect is intensified among students since this demographic is also heavily affected by a multitude of other stressors, including poor work–life balance and worsened mental health, which may heighten academic pressure [74]. Moreover, students with higher GAS scores are more likely to experience poor sleep quality and daytime sleepiness, amplifying academic stress and stimulant reliance [75,76].

Given that problematic gaming is an emerging issue that was positively associated with NMPDU in this sample, clinicians may consider evaluating gaming addiction symptoms as part of routine mental health screenings for young adults [77]. Providers might assess psychological constructs that are commonly associated with IGD, such as withdrawal, impulsivity, and hostility [78]. The 7-item Gaming Addiction Scale (GAS) may serve as a valid and reliable tool to identify individuals at risk for gaming addiction [42]. Screening protocols could be particularly relevant for students and other populations who demonstrated a stronger association between GAS and NMPDU. Existing screening questionnaires, such as the Drug Abuse Screening Test (DAST), may be implemented by clinicians and/or colleges to identify individuals at risk for NMPDU (DAST) [79]. For individuals with gaming addiction, interventions including craving behavioral intervention (CBI), cognitive behavior therapy (CBT), and group counseling may offer greater control over gaming addiction symptoms and reduce adverse effects [80]. These interventions target underlying motivations for problematic gaming by diminishing sensitivity to gaming-related cues, allowing individuals to recognize and challenge maladaptive thoughts, and providing peer support [80].

Among student populations, universities could develop initiatives that educate students about problematic gaming and its relationship to NMPDU, emphasizing stress management, gaming-based media literacy, and the risks of NMPDU [81]. University-based programs that allow safe environments for open discussions can establish positive peer networks and reduce social isolation, which is a significant risk factor for both conditions [82]. Institutions may also consider training mental health counselors to recognize students at risk for IGD, establishing a more targeted approach to addressing these issues [82].

### Limitations

This study is not without limitations. First, this is a cross-sectional study and causality cannot be inferred. The GAS assesses symptoms over the prior six months, whereas NMPDU was measured as lifetime use. This temporal mismatch limits our ability to determine whether problematic gaming preceded or followed nonmedical prescription drug use. Accordingly, results should be interpreted as associative rather than directional. Furthermore, the lifetime NMPDU measure does not distinguish between sporadic and recurrent nonmedical use, nor between drug classes (e.g., stimulants, opioids, benzodiazepines). This may obscure important heterogeneity in risk patterns. Future studies should incorporate time-aligned, substance-specific, and frequency-based measures to better clarify temporal and behavioral pathways. This is also a self-report study, so behaviors may be subject to social desirability bias, like under-reporting NMPDU. However, any bias is likely at random in respect to GAS score and should not impact study findings. Recall bias may be a concern, and NMPDU is likely under-reported. Although single-item self-report measures of nonmedical prescription drug use are common in surveillance research and have demonstrated acceptable test–retest reliability in youth risk behavior contexts, measurement error and heterogeneity in misuse patterns remain possible [39,40,83]. The RIYAS employed a non-probability convenience sampling strategy using targeted social media advertisements and online recruitment. As such, the sample may not be representative of all Rhode Island young adults, and selection bias is possible. Individuals who are more engaged online or more interested in mental health topics may have been more likely to participate. However, social media recruitment has been shown to be an effective strategy for reaching diverse young adult populations and is commonly used in behavioral health surveillance research [84]. Additionally, participants received a modest $10 incentive for survey completion. While financial incentives are widely used to enhance survey participation, they may influence who elects to participate, potentially introducing additional participation bias. Furthermore, as this study is composed of young adults from a small Northeastern state, generalizability of these results to other populations may be limited. Therefore, findings should be interpreted as associative relationships within a convenience sample rather than population-representative estimates. Finally, ADHD, a potential confounder, was not measured or controlled for—residual confounding is plausible.

## 5. Conclusions

In this study of Rhode Island young adults, NMPDU was prevalent and students with higher GAS scores demonstrated a stronger association with NMPDU compared to their non-student counterparts. Screening protocols may be adapted to assess gaming addiction symptoms and target populations at a higher risk for NMPDU. Providers may consider craving behavioral intervention (CBI), cognitive behavior therapy (CBT), and group counseling as viable treatment methods for gaming addiction. In universities, interventions that create positive peer-based connections and provide students with greater stress management resources may be beneficial.

## Figures and Tables

**Table 1:** Characteristics of Rhode Island Young Adults by Nonmedical Prescription Drug Use (NMPDU).

	Total N = 1022 (%)	NMPDU N = 124 (12.1%)	No NMPDU N = 898 (87.9%)	*p*-Value
Age [Mean (SE)]	21.3 (0.07)	21.6 (0.21)	21.3 (0.07)	0.108
Sex, Gender, and Sexuality				0.119
Cisgender Heterosexual Male	133 (13.0)	15 (12.1)	118 (13.1)	
Cisgender Heterosexual Female	456 (44.6)	46 (37.1)	410 (45.7)	
Sexual and Gender Minority	433 (42.4)	63 (50.8)	370 (41.2)	
Race/Ethnicity				0.302
White, non-Hispanic	623 (61.0)	75 (60.5)	548 (61.0)	
Black, non-Hispanic	56 (5.5)	4 (3.2)	52 (5.8)	
Asian, non-Hispanic	62 (6.1)	7 (5.7)	55 (6.1)	
Hispanic	212 (20.7)	33 (26.6)	179 (19.9)	
Other/Multiracial, non-Hispanic	69 (6.8)	5 (4.0)	64 (7.1)	
Social Status [Mean (SE)]	6.0 (0.05)	6.0 (0.16)	6.0 (0.06)	0.731
AUDIT [Mean (SE)]	4.0 (0.14)	6.1 (0.52)	3.7 (0.15)	<0.001
CUDIT-R [Mean (SE)]	3.6 (0.18)	6.4 (0.68)	3.2 (0.18)	<0.001
Depressive Symptoms [Mean (SE)]	10.4 (0.21)	13.1 (0.62)	10.0 (0.22)	<0.001
Anxious Symptoms [Mean (SE)]	8.31 (0.19)	10.5 (0.56)	8.00 (0.20)	<0.001
Gaming Addiction Scale [Median (IQR)]	7 (4)	7 (7.5)	7 (4)	0.018
Student				0.053
Yes	719 (70.4)	78 (62.9)	641 (71.4)	
No	303 (29.7)	46 (37.1)	257 (28.6)	

**Table 2:** Adjusted Odds of Nonmedical Prescription Drug Misuse (NMPDU) Among Rhode Island Young Adults, N = 1022.

					NMPDU	
	B	SE	Wald	*p*	AOR	95% CI
Age	0.06	0.05	1.21	0.273	1.06	0.95–1.18
Sex, Gender, and Sexuality						
Cisgender Heterosexual Male	Ref				1.00	
Cisgender Heterosexual Female	0.23	0.35	0.44	0.512	1.26	0.63–2.53
Sexual and Gender Minority	0.23	0.34	0.46	0.499	1.26	0.65–2.46
Race/Ethnicity						
White, non-Hispanic	Ref				1.00	
Black, non-Hispanic	−0.71	0.58	1.51	0.218	0.49	0.16–1.53
Asian, non-Hispanic	0.13	0.44	0.08	0.769	1.14	0.48–2.72
Hispanic	0.49	0.24	3.96	0.046	1.63	1.01–2.63
Other/Multiracial, non-Hispanic	−0.53	0.50	1.14	0.284	0.59	0.22–1.56
Social Status	−0.10	0.06	2.89	0.089	0.90	0.80–1.02
AUDIT	0.06	0.02	8.47	0.004	1.06	1.02–1.10
CUDIT-R	0.04	0.02	7.62	0.006	1.04	1.01–1.08
Depressive Symptoms	0.04	0.02	2.43	0.120	1.04	0.99–1.09
Anxious Symptoms	0.01	0.03	0.30	0.580	1.01	0.96–1.07
Gaming Addiction Scale	0.05	0.02	5.76	0.017	1.05	1.01–1.09
Student	−0.22	0.23	0.92	0.337	0.80	0.51–1.26
Interaction Model						
GAS x Student	0.09	0.04	4.62	0.031	1.09	1.01–1.18

Note: Model without interaction: χ^2^(14, N = 1022) = 66.78, *p* < 0.001, Nagelkerke R^2^ = 0.11; Model with interaction: χ^2^(15, N = 1022) = 71.75, *p* < 0.001, Nagelkerke R^2^ = 0.12.

## Data Availability

Data can be made available by reasonable request to the Rhode Island Department of Behavioral Healthcare, Developmental Disabilities & Hospitals.
